# Evidence for the association between *FTO* gene variants and vitamin B12 concentrations in an Asian Indian population

**DOI:** 10.1186/s12263-019-0649-3

**Published:** 2019-09-05

**Authors:** Shelini Surendran, Ramamoorthy Jayashri, Lauren Drysdale, Dhanasekaran Bodhini, Nagarajan Lakshmipriya, Coimbatore Subramanian Shanthi Rani, Vasudevan Sudha, Julie A. Lovegrove, Ranjit M. Anjana, Viswanathan Mohan, Venkatesan Radha, Rajendra Pradeepa, Karani S. Vimaleswaran

**Affiliations:** 1 Hugh Sinclair Unit of Human Nutrition and Institute for Cardiovascular and Metabolic Research (ICMR), Department of Food and Nutritional Sciences, University of Reading, Whiteknights, PO Box 226, Reading, RG6 6AP UK; 2 Department of Diabetology, Madras Diabetes Research Foundation and Dr. Mohan’s Diabetes Specialities Centre, WHO Collaborating Centre for Non-communicable Diseases Prevention and Control, ICMR Centre for Advanced Research on Diabetes, Gopalapuram, Chennai, 600086 India; 3 School of Psychology and Clinical Language Sciences, University of Reading, Reading, UK; 4 Department of Molecular Genetics, Madras Diabetes Research Foundation, Chennai, India; 5 Department of Foods, Nutrition and Dietetics Research, Madras Diabetes Research Foundation, Chennai, India; 6 Department of Clinical Epidemiology, Madras Diabetes Research Foundation, Chennai, India

**Keywords:** SNP, Obesity, Metabolic traits, Vitamin B12 pathway, South Asian, Indian, Nutrigenetics

## Abstract

**Background:**

Low vitamin B12 concentrations have been associated with major clinical outcomes, including adiposity, in Indian populations. The Fat mass and obesity-associated gene (*FTO*) is an established obesity-susceptibility locus; however, it remains unknown whether it influences vitamin B12 status. Hence, we investigated the association of two previously studied *FTO* polymorphisms with vitamin B12 concentrations and metabolic disease-related outcomes and examined whether these associations were modified by dietary factors and physical activity.

**Methods:**

A total of 176 individuals with type 2 diabetes, 152 with pre-diabetes, and 220 normal glucose-tolerant individuals were randomly selected from the Chennai Urban Rural Epidemiology Study. Anthropometric, clinical, and biochemical investigations, which included body mass index (BMI), waist circumference, vitamin B12, homocysteine, and folic acid were measured. A validated food frequency questionnaire was used for dietary assessment and self-reported physical activity measures were collected. An unweighted genetic risk score (GRS) was calculated for two *FTO* single-nucleotide polymorphisms (rs8050136 and rs2388405) by summation of the number of risk alleles for obesity. Interaction analyses were performed by including the interaction terms in the regression model.

**Results:**

The GRS was significantly associated with increased BMI (*P* = 0.009) and risk of obesity (*P* = 0.023). Individuals carrying more than one risk allele for the GRS had 13.13% lower vitamin B12 concentrations, compared to individuals carrying zero risk alleles (*P* = 0.018). No associations between the GRS and folic acid and homocysteine concentrations were observed. Furthermore, no statistically significant GRS-diet or GRS-physical activity interactions with vitamin B12, folic acid, homocysteine or metabolic-disease outcomes were observed.

**Conclusion:**

The study shows for the first time that a genetic risk score using two *FTO* SNPs is associated with lower vitamin B12 concentrations; however, we did not identify any evidence for the influence of lifestyle factors on this association. Further replication studies in larger cohorts are warranted to investigate the association between the GRS and vitamin B12 concentrations.

**Electronic supplementary material:**

The online version of this article (10.1186/s12263-019-0649-3) contains supplementary material, which is available to authorized users.

## Introduction

Obesity and its related comorbidities are leading causes of mortality and morbidity worldwide [1]. It is estimated that > 12% of the Indian population is either overweight or obese [2]. Epidemiological studies have documented that the increased accessibility of low-cost, high-calorie, and nutrient-poor foods was among the major driving forces for the epidemic of obesity [3–5]. This has led to a substantial increase in the prevalence of obesity-associated metabolic problems, such as type 2 diabetes mellitus (T2DM), dyslipidemia, and hypertension in India [6]. Furthermore, several studies have also demonstrated that obesity is associated with substantial nutrient deficiencies, including vitamin B12 [7–9].

Vitamin B12 deficiency is a major public health problem in India and a recent cross-sectional study conducted in 630 healthy adults in a South Indian population, reported that 35% of adults were vitamin B12 deficient [10]. An adequate vitamin B12 concentration is essential for growth, development, and health. In addition, it is essential for DNA synthesis, hematological development, and maintenance of the myelin nerve sheaths [11–13]. The primary causes of vitamin B12 deficiency are age, consumption of vegetarian diets, and the inability to absorb vitamin B12 from food (via genetic defects or disease) [14, 15]. To date, several studies have indicated that vitamin B12 status may be influenced by excess body weight [16, 17]. However, a recent pooled analysis of 19 studies found no evidence for an inverse relationship between vitamin B12 and BMI levels and reported that the majority of observational studies had a high risk of bias and heterogeneity due to the fact that most of the studies were not designed to investigate the association between B12 level and BMI [18]. In light of these findings, using a genetic approach to explain the genetic mechanisms for obesity and its link with vitamin B12 concentrations could be a better option, in terms of reducing any influence from unmeasured confounding factors.

Genome-wide association studies have identified several genetic variants related to obesity and type 2 diabetes risk [19, 20]. To date, the fat mass and obesity-associated (*FTO*) gene has been identified as the strongest common genetic predictor of obesity [21]. Individuals who are homozygous for *FTO* risk alleles are on average at 1.67-fold increased odds of obesity and 3 kg heavier in comparison to individuals without any risk alleles [22]. While several studies have reported the association between the *FTO* gene on measures of body weight and composition, various dietary parameters and physical activity levels have also been shown to contribute [23–25]. Recently, a cross-sectional study in an Indian population showed that physical activity and dietary intake may modify the association between the *FTO* gene variants and obesity-related traits [26]. We used *FTO* gene variants as instruments to establish the relationship between obesity and B12 status and tested whether this relationship was modified by lifestyle factors. The two main objectives of this study were first to determine whether the *FTO* single-nucleotide polymorphisms (SNPs), rs8050136, and rs2388405, were associated with obesity traits, vitamin B12, folic acid, and homocysteine and secondly whether these associations were modified by diet and physical activity levels in Asian Indians.

## Methodology

### Study population

**Fig. 1:**
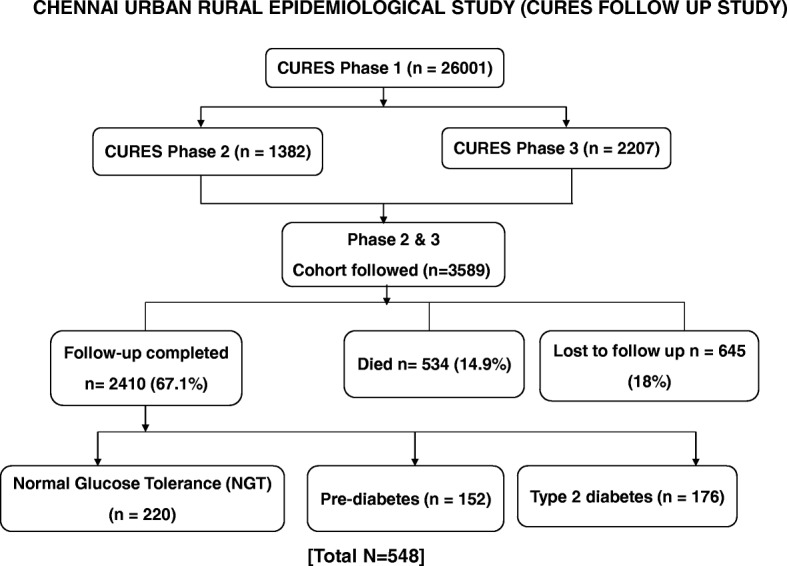
Flow diagram describing the selection of study participants

A total of 548 unrelated study subjects were randomly recruited from the Chennai Urban Rural Epidemiology Study (CURES) follow-up study, which is an epidemiological study conducted on a representative population of Chennai, (formerly Madras) in southern India. The methodology of the study is published elsewhere [27, 28] and is briefly outlined here (Fig. 1). In phase 1 of CURES, 26,001 (aged ≥ 20 years) individuals were recruited based on a systematic random sampling technique. In the baseline survey, of the 26,001 individuals screened, all the individuals with diabetes (phase 2, *n* = 1382) and 1 in every 10 individuals (phase 3, *n* = 2207) underwent further detailed investigations, and these constituted the cohort for the follow-up study (*n* = 3589). From these 3589 individuals, 548 individuals, which included: 220 NGT, 152 prediabetic, and 176 T2DM individuals were randomly selected for this study. Individuals were excluded from participation if they were known cases of type 1 diabetes, had diabetes secondary to other causes, e.g., chronic pancreatitis, if they were 80 years of age, or were taking vitamin B12 supplements. Table 1 shows the characteristics of the study participants.

**Table 1:** Baseline characteristics of the CURES study participants: comparison of non-obese and obese individuals

Characteristic	*n*	Non-obese individuals	*n*	Obese individuals	*P* value*
Age (years)	194	51.0 ± 12.8	353	48.4 ± 10.4	0.017
BMI (kg/m^2^)	194	21.9 ± 2.1	353	29.4 ± 4.1	< 0.001
WC (cm)	194	79.7 ± 8.1	352	93.0 ± 9.9	< 0.001
Hip (cm)	194	89.1 ± 6.1	352	102.4 ± 9.4	< 0.001
WHR	194	0.90 ± 0.08	352	0.91 ± 0.08	0.061
Fasting plasma glucose (mg/dl)	184	119.5 ± 60.3	335	114.7 ± 41.6	0.336
Fasting serum insulin (μIU/ml)	194	8.75 ± 6.36	353	9.57 ± 5.92	0.132
Glycated hemoglobin (%)	194	6.5 ± 1.9	353	6.5 ± 1.6	0.916
Vitamin B12 levels (pg/mL)	194	414.0 ± 219.0	353	420.0 ± 273.0	0.820
Homocysteine	194	14.1 ± 8.2	353	13.4 ± 8.0	0.343
(μmol/L)					
Folic acid	194	8.9 ± 5.6	353	8.5 ± 5.9	0.451
(ng/ml)					
Total energy intake (kcal)	129	2547.0 ± 784.0	234	2624.0 ± 768.0	0.363
Protein energy %	129	11.3 ± 1.2	234	11.3 ± 1.1	0.947
Carbohydrate energy %	129	63.3 ± 6.7	234	64.9 ± 6.0	0.020
Fat energy %	129	24.2 ± 4.6	234	23.6 ± 4.8	0.293
Total fiber (g)	129	31.6 ± 10.2	234	32.5 ± 11.9	0.513
Physical activity level	123	Low (81.3%)	204	Low (82.4%)	0.929^a^
		Medium (17.1%)		Medium (15.7%)	
		High (1.6%)		High (2.0%)	

Data shown are represented as means ± SD

*P* values were calculated by using the Independent *t* test

*P values for the differences in the means/proportions between non-obese and obese individuals

^a^*P* values were calculated by using the chi-squared test

Abbreviations: *CURES* Chennai Urban Rural Epidemiological Study; *BMI* body mass index; *WC* waist circumference; *WHR* waist to hip ratio

The Madras Diabetes Research Foundation Institutional Ethics Committee granted ethical approval, and informed consent was obtained from the study participants. All clinical investigations were conducted according to the principles expressed in the Declaration of Helsinki (ICH GCP).

### Phenotype measurements

Anthropometric measurements including weight, height, and waist circumference were measured using standardized techniques. The body mass index (BMI) was calculated using the formula, weight (kg)/height (m^2^), and obesity was classified as BMI ≥ 25 according to WHO Asia Pacific Guidelines for Asians (The Asia Pacific perspective 2000). Fasting plasma glucose (glucose oxidase–peroxidase method) was measured using Hitachi-912 Autoanalyser (Hitachi, Mannheim, Germany). Glycated hemoglobin (HbA1c) was estimated by high-performance liquid chromatography using a Variant™ machine (Bio-Rad, Hercules, CA, USA). Serum insulin, serum vitamin B_12_, and folic acid concentration were estimated using the electrochemiluminescence using a Roche e601Cobas immunoassay analyzer (Roche Diagnostics, Indianapolis, IN, USA). The intra- and inter-assay coefficients of variation for vitamin B_12_ assay were 0.95% and 4.08%. Serum homocysteine was measured using enzymatic assay using the Beckman Coulter AU2700 (Fullerton, CA, USA) Biochemistry analyzer.

### Dietary assessments and physical activity

Dietary intakes were assessed using a previously validated and published interviewer-administered semi-quantitative food frequency questionnaire (FFQ) containing 222 food items to estimate food intake over the past year. The length of the interview ranged from 20 and 30 min during which participants were asked to recall their usual portion size and usual frequency (number of times per day, week, month, or year/never) of foods listed within the FFQ over the year. Common household measures such as household cups, bowls, ladles, spoons (for the cooked foods like vegetables), wedges, circles of different diameter and visual atlas of different sizes of fruits (small, medium, large) were shown to assist the individuals in estimating portions. A detailed description of the development of FFQ and the data on reproducibility and validity had been published previously [29]. The recorded data was analyzed with the EpiNu® software to estimate energy as well as macronutrient and dietary fiber intake.

A validated self-report questionnaire was used to measure physical activity questionnaire [30]. Based on exercise, leisure time activities, and job-related activities, respondents were categorized into three groups indicating activity level (vigorously active, moderately active, and sedentary). Individuals were graded as vigorously active if they did leisure-time exercise and had physically demanding work, whereas individuals who either exercised or had physically demanding work were categorized as moderately active. All others were categorized as sedentary.

### SNP selection and genotyping

Genetic variants within the *FTO* gene have shown consistent and strong associations with obesity [21]. Evidence suggests that the *FTO* gene confers an increased risk of obesity by approximately 1.20-fold, and a corresponding increase in BMI by 0.39 kg/m^2^ per minor allele [31]. The BMI-increasing allele in the *FTO* gene is less prevalent in Asian (~ 30%) and African populations (~ 12%) than in European ancestry populations (~ 42%). However, the effect of the risk alleles on BMI variance is somewhat similar in the Asian (0.2%), African (0.1%) and European populations (0.3%) [31–33].

Of particular interest are intronic SNPs, which may harbor ‘intronic enhancers’ that may exert functional effects and contain potential transcriptional factor binding sites. Furthermore, some of these intronic variants have been shown to increase disease risk or modulate the genotype-phenotype relationship [34]. The SNP rs8050136 of the *FTO* gene has shown consistent and strong associations with obesity and type 2 diabetes [21]. Additionally, the SNP rs2388405 was previously selected for analysis in a case-control study conducted in a Chinese population, due to its possibility of being an ‘intronic enhancer’ [35] and also in a study in a Han Chinese population [36] and a Caucasian population [37]. Hence, we selected these two intronic SNPs of the *FTO* gene with a known minor allele frequency (MAF) > 15% in the South Asian population: rs8050136 (intron 1, MAF = 29%; HapMap South Asian population) and rs2388405 (intron 4, MAF = 40%; HapMap South Asian population).

The standard Phenol-chloroform method was used to extract DNA from whole blood [38]. The SNPs rs8050136 and rs2388405 were genotyped by polymerase chain reaction on a GeneAmp PCR system 9700 thermal cycler (Applied Biosystems, Foster City, CA) using the primers “F: 5′TTT GTT TTG GCT TTC TGC AGT CT3′, R: CAA AAA CCA CAG GCT CAG A3′ and F: 5′TCT GTG GGA ATC TCC GCT TTC AGT, R: 5′GAG CCC TTG CGC ATT GCC AG3′ respectively. The PCR products were digested with MluCI (rs8050136) and ScaI (rs2388405) restriction enzymes (New England Biolabs, Inc., Beverly, MA) and the digested products were resolved by a 3% agarose gel electrophoresis. Based on the analysis of 200 blind duplicates (20%), there was 100% concordance in the genotyping. Furthermore, a few variants were confirmed by direct sequencing with an ABI 310 genetic analyzer (Foster City, CA).

### Statistical analysis

The SPSS statistical package (version 22; SPSS Inc., Chicago, IL, USA) was used for the statistical analysis. Allele frequencies were estimated by gene counting. The chi-square test was used to compare the proportions of genotypes or alleles. The genotypic frequencies in all participants showed no significant departure from the Hardy Weinberg Equilibrium (HWE) (*P* > 0.05) for the *FTO* rs8050136 (MAF 0.13 and HWE *P* = 0.749) and rs2388405 (MAF 0.09 and HWE *P* = 0.259) SNPs.

Generalized obesity was defined according to the World Health Organization Asia Pacific Guidelines for Asians as non-obese (BMI < 25 kg/m^2^ ) and obese (BMI ≥ 25 kg/m^2^) [39]. We performed an independent *t* test to compare the means of the quantitative variables between individuals with normal-glucose tolerance (NGT) vs pre-diabetes and NGT vs T2D). Comparison of the proportion of individuals engaging in different types of physical activity levels (vigorously active, moderately active, and sedentary) between NGT individuals vs pre-diabetes and NGT individuals vs T2D was analyzed by the chi-square test.

The unweighted, risk-allele GRS method was calculated for each participant by summation of the number of risk alleles for obesity. The GRS was generated from the SNPs rs8050136 and rs2388405 of the *FTO* gene. A value of 0, 1, or 2 was assigned to each SNP, which denotes the number of risk alleles for obesity on that SNP. These values were then calculated by adding the number of risk alleles across each SNP. The risk allele score was then divided into individuals carrying 0 risk allele vs more than 1 risk alleles. Association analyses between the GRS and continuous and categorical variables were carried out by linear and logistic regression models, respectively. Linear and logistic regression models were also used for interaction analyses between GRS and dietary factors (continuous variables)/physical activity (categorical variable) on continuous and categorical outcomes respectively, where the interaction terms were included in the models and were adjusted for age, BMI, sex, T2D, T2D medication, and total energy intake when appropriate.

Correction for multiple testing was applied using Bonferroni correction [adjustment *P* value for association analysis was < 0.0083 [1 GRS × 6 biochemical and metabolic traits (vitamin B12, Homocysteine, folic acid, obesity, BMI, waist circumference) = 6 tests)] and for interaction < 0.0017 [1 GRS × 6 biochemical and metabolic traits × 5 lifestyle factors (dietary carbohydrate energy %, dietary protein-energy %, dietary fat energy %, dietary fiber intake (g), and physical activity levels) = 30 tests]. Given that there are no studies on GRS and no previously reported effect sizes for the South Asians, we were unable to perform a power calculation for the present study.

## Results

### Characteristics of the participants

The clinical and biochemical characteristics of the individuals from the CURES study are illustrated in Table 1. No significant difference between obese and non-obese individuals were observed in the levels of fasting glucose, insulin, HbA1c, folic acid, homocysteine, vitamin B12 and waist to hip ratio (P>0.05). However we observed that obese individuals consumed higher quantities of dietary carbohydrate (energy %) than non-obese individuals (P = 0.020). The baseline characteristics which compares individuals with NGT, pre-diabetes, and T2D is shown in Additional file 1: Table S1.

### Association of B12 level with prediabetes and type 2 diabetes

After adjusting for age, sex, and BMI, there was no association of vitamin B12 level with prediabetes (*P* = 0.19) and type 2 diabetes (*P* = 0.52). Likewise, there was no association of vitamin B12 level with prediabetes (*P* = 0.22) and type 2 diabetes (*P* = 0.57) after adjusting for age, sex, and GRS (as an instrument for BMI).

### Association between GRS and obesity-related phenotypes

**Fig. 2:**
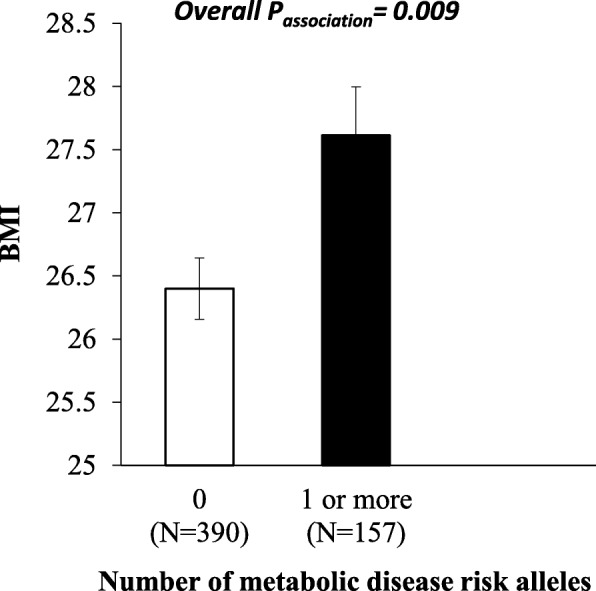
Association between the GRS and BMI

We were able to identify an association between GRS and BMI (*P* = 0.009). Individuals who carried more than one risk allele had higher BMI levels (mean ± SD: 27.55 ± 4.98) compared to individuals with zero risk alleles (mean ± SD: 26.43 ± 5.03) (Table 2 and Fig. 2).

**Table 2:** Association between the *FTO*-GRS with vitamin B12, folic acid, homocysteine, and obesity traits

Risk alleles	*n*	Vitamin B12 (pg/mL)	*n*	Homocysteine (μmol/L)	*n*	Folic acid (ng/ml)	*n*	BMI (kg/m^2^)	*n*	WC (cm)	*n*	Odds Ratio (95% CI) of Obesity
**0**	380	410 ± 202	390	13.2 ± 7.7	390	8.89 ± 5.92	390	26.4 ± 5.0	390	87.6 ± 11.1	194	1.63 (1.07-2.49)
≥ 1	154	356 ± 189	157	14.8 ± 8.9	157	7.89 ± 5.48	157	27.6 ± 5.0	156	90.0 ± 11.6	353	
*P* value	0.018		0.077		0.147		0.009†		0.747		*0.023	

Values are given as mean ± standard deviation

*P* values for differences between 0 and 1 risk alleles were obtained using linear regression model adjusted age, BMI, type 2 diabetes status, type 2 diabetes medication, and sex

†*P* values were obtained by using a general linear model adjusted for age, type 2 diabetes status, type 2 diabetes medication, and sex

*P values were adjusted for age, sex, and type 2 diabetes status using binary logistic regression

Abbreviations: *BMI* body mass index; *WC* waist circumference; *WHR* waist to hip ratio

Obesity risk-increasing alleles ranged from 0 to 3. The white bars indicate individuals with 0 risk alleles and the black bars indicate individuals carrying ≥ 1 alleles. Individuals who carried 1 or more risk alleles had significantly higher BMI compared to individuals carrying 0 risk alleles (*P* = 0.009)]

There was a significant association between the GRS and obesity (*P*_association_ = 0.023), where individuals carrying more than one risk allele had 1.6 times increased risk of obesity compared to those carrying zero risk alleles (Table 2). However, after Bonferroni correction, none of these associations remained statistically significant. Moreover, no statistically significant associations were observed between GRS and waist circumference (*P* = 0.747) (Table 2).

### Association between the GRS and vitamin B12, homocysteine, and folic acid levels

**Fig. 3:**
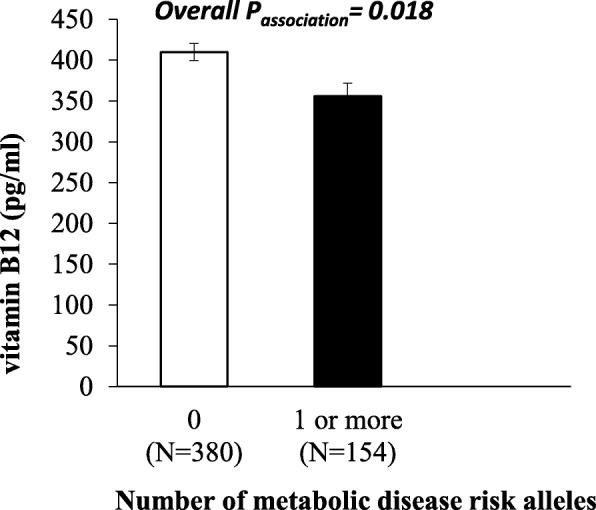
Association between the GRS and serum vitamin B12 levels

We found that the GRS was significantly associated with vitamin B12 concentrations (*P* = 0.018) (Table 2 and Fig. 3), and individuals carrying more than one risk allele had 13.1% lower vitamin B12 concentrations (mean ± SD: 355 ± 189 pg/mL), compared to individuals carrying zero risk alleles (mean ± SD: 410 ± 202 pg/mL). However, this finding was not significant after correction for multiple testing.

Obesity risk-increasing alleles ranged from 0 to 3. The white bars indicate individuals with 0 risk alleles and the black bars indicate individuals carrying ≥ 1 alleles. Individuals who carried 1 or more risk alleles had significantly lower B12 concentrations compared to individuals carrying 0 risk alleles (*P* = 0.018)] (Fig. 3).

There were no statistically significant associations between GRS and homocysteine or folic acid concentrations (Table 2).

### Interaction between the GRS and lifestyle factors on vitamin B12, folic acid, homocysteine and obesity traits

None of the lifestyle factors (dietary intake (carbohydrate, protein, fat, fiber) or physical activity) significantly interacted with the GRS on biochemical and anthropometric measurements after correction for multiple testing (Additional file 2: Table S2 and Additional file 3: Table S3).

## Discussion

Both obesity and vitamin B12 deficiency are modifiable risk factors for several chronic diseases. Moreover, both risk factors have been shown previously to be associated with one another. This is the first study to use a genetic approach to establish a relationship between obesity and vitamin B12 levels in an Asian Indian population. Our study confirmed the strength of the association between the GRS generated from the two *FTO* SNPs and BMI and demonstrated the impact of genetically instrumented BMI on serum B12 concentrations. These results suggest that increases in BMI could potentially contribute to the adverse health effects associated with vitamin B12 deficiency. Given that low vitamin B12 concentrations in Asian Indians are common [10, 40], our study highlights the importance of considering obesity as a risk factor for vitamin B12 deficiency with implications on the possible targeting of relevant obesity prevention strategies.

Variants of the *FTO* gene are known to be the strongest genetic predictor of obesity to date [41, 42]. It has been suggested that risk variants at the *FTO* locus trigger the overexpression of ghrelin mRNA, leading to higher levels of the hunger hormone, ghrelin, to be secreted [43], which in turn makes individuals over consume energy-dense foods [44, 45]. In general, the two selected intronic SNPs rs2388405 and rs8050136 could potentially be relevant as intronic enhancers, as they may enhance the expression of the *FTO* gene [34]. In support of this, in a previous study conducted in a South Indian population (CURES), the *FTO* SNP, rs8050136, was associated with an increased risk of obesity [46]. Given the strong role of the *FTO* locus in obesity [21, 46], *FTO* was considered as a suitable candidate to establish the genetic link between obesity-related traits and vitamin B12 concentrations.

Reduced vitamin B12 concentrations in the obese population are thought to result from a nutrient-poor diet, increased nutrient requirements in relation to increased body size and the physiological effects of obesity on nutrient absorption/metabolism [47, 48]. Additionally, obesity is a well-known risk factor for T2DM [49] and gastroesophageal reflux disease (GERD) [50]. As a result, obese individuals are more likely to take metformin and proton pump inhibitors (PPIs), which have been shown to reduce serum B12 levels by inhibiting the absorption of the vitamin [51, 52]. However, two large Mendelian randomization studies in populations of European ancestry failed to confirm a causal relationship between low vitamin B12 concentrations and increased BMI [53, 54]. In our study, we found a significant association of the *FTO* GRS (increased BMI) with low vitamin B12 concentrations in South Asian adults. Several studies in India have reported significant phenotypic associations between vitamin B12 status and obesity-related traits [9, 16, 55, 56]. A study conducted in North India reported that there was a negative correlation between waist circumference and reduced levels of vitamin B12 [55]. A study looking at 2403 school-going adolescents (11–17 years) from Haryana, India reported that more than half (51.2%) of obese adolescents were vitamin B12 deficient [9]. Furthermore, recent findings from the CURES (*n* = 1500 individuals) demonstrated that the prevalence of vitamin B12 deficiency significantly increased in those with abdominal obesity and the mean levels of vitamin B12 significantly decreased with increasing degrees of glucose tolerance [56]. However, in this study, we were unable to identify a similar trend when considering the GRS, which could be due to the smaller sample size of our study (data not shown). However, our data in Asian Indians confirms the association between vitamin B12 concentrations and obesity and suggests that individuals genetically predisposed to obesity are at a higher risk of vitamin B12 deficiency. Bi-directional Mendelian randomization studies examining the causal relationship between B12 level and obesity should be examined in Europeans and Asian Indians to identify ethnic specific differences.

Current literature suggests that the genetic profile of an individual can shape the microbiome of the host, and indeed an altered gut flora has been associated with vitamin B12 deficiency [15, 57]. In a study in rodents, it was found that the type of dietary lipids (lard or fish oil) influenced the structure of the microbiome as there was an interaction between gut microbiota and saturated lipids in promoting white adipose tissue inflammation [58]. Chakraborty et al. postulated that a higher concentration of inflammatory cytokines could impair vitamin B12 absorption or biosynthesis [9]. Another study reported that low vitamin B12 status induced excess triacylglycerol biosynthesis and secretion of pro-inflammatory cytokines [59]. Whether the *FTO* genotypes influence the association between obesity and vitamin B12 concentrations by modulating the gut microbiota composition and inducing metabolic inflammation requires further investigation utilizing fecal samples.

The main strength of this study was the use of a validated food frequency questionnaire [60], which has shown high reproducibility and validity for total carbohydrates and dietary fiber, and the use of a GRS. Moreover, the sampling was representative of the overall population of Chennai. Nevertheless, some limitations need to be acknowledged. Although the majority of Indian adults are physically inactive and consume a diet high in carbohydrates [23, 61], no significant interactions were found between the GRS and lifestyle factors on vitamin B12 and metabolic disease outcomes in our study, which could be attributed to the small sample size. The GRS only used two variants from the *FTO* gene, and we cannot fully exclude that other variants of the *FTO* gene may also be important. Furthermore, previous studies have shown an association of B12 level with pre-diabetes and T2D [62–64]; hence, it is possible that the genetic associations identified in this study could have been mediated through the association of B12 level with diabetes. But, after adjustment for BMI, there was no significant association of B12 level with pre-diabetes and T2D suggesting the effect of GRS on B12 level in Asian Indians. Another limitation was the use of a cross-sectional design to investigate genetic effects at a single point in time and hence no cause-effect inferences can be drawn, for which a longitudinal analysis design over a specific time period would be needed.

In summary, our study, for the first time, suggests that genetic variations at the *FTO* locus appear to influence serum vitamin B12 concentrations in Asian Indians. However, we were unable to show an impact of the GRS on lowering B12 concentrations through a dietary influence. Longitudinal studies and large bi-directional Mendelian randomization studies could help to establish the causal relationship between vitamin B12 status and obesity in Asian Indians.

## Additional files

Additional file 1: **Table S1.** Baseline characteristics of the CURES study participants: Comparison of NGT, Pre-diabetics and T2D individuals. (PDF 108 kb)

Additional file 2: **Table S2.** Interaction between the *FTO*-GRS and lifestyle factors on vitamin B12, folic acid, homocysteine and obesity traits. (PDF 96 kb)

Additional file 3: **Table S3.** Interaction between the *FTO*-GRS and dietary factors on obesity. (PDF 33 kb)

## Data Availability

The datasets used during the current study are available from the corresponding author on reasonable request.

## Abbreviations

BMI: Body mass index
FTO: Fat mass and obesity-associated
GRS: Genetic risk score
SD: Standard deviations
SNPs: Single-nucleotide polymorphisms
WC: Waist circumference
WHR: Waist to hip ratio
