# Supplementary material for: Evidence for the association between FTO gene variants and vitamin B12 concentrations in an Asian Indian population
Source: Genes Nutr. 2019 Sep 5;14:26. doi: 10.1186/s12263-019-0649-3 (PMC6728975; doi:10.1186/s12263-019-0649-3)
Supplement: Supplementary file 1 — Table S1. Baseline characteristics of the CURES study participants: Comparison of NGT, Pre-diabetics and T2D individuals. (PDF 108 kb) [file 12263_2019_649_MOESM1_ESM.pdf]

Supplementary Table 1: Baseline characteristics of the CURES study participants: Comparison of NGT, Pre-diabetics and T2D individuals

Characteristic	n	Participants with normal glucose tolerance (NGT)	n	Pre-diabetics	P value *	n	Participants with type 2 diabetes (T2D)	P value **
Age (yrs)	300	48.2 ± 11.9	300	48.4 ± 11.7	0.849	300	53.6 ± 11.0	<0.001
BMI (kg/m^2)	300	25.8 ± 5.0	300	27.4 ± 5.2	<0.001	299	26.9 ± 4.6	0.010
WC (cm)	300	85.9 ± 11.4	300	89.6 ± 11.1	<0.001	298	90.3 ± 10.1	<0.001
Hip (cm)	300	96.4 ± 11.0	300	98.7 ± 11.6	0.015	298	97.6 ± 9.6	0.174
WHR	300	0.89 ± 0.09	300	0.91 ± 0.09	0.022	298	0.93 ± 0.08	<0.001
Fasting plasma glucose (mg/dl)	274	90 ± 6	283	103 ± 18	<0.001	296	154 ± 61	<0.001

Fasting serum insulin (µIU/ml)	300	8.3 ± 5.6	300	8.1 ± ± 5.6	0.757	300	11.3 ± 6.9	<0.001
Glycated Haemoglobin (%)	300	5.7 ± 0.6	300	5.9 ± 0.6	<0.001	300	8.1 ± 2.0	<0.001
Vitamin B12 levels (pg/mL)	300	450 ± 332	300	409 ± 246	0.086	300	389 ± 211	0.008
Homocysteine (µmol/L)	300	13.1 ± 6.1	300	13.3 ± 7.4	0.650	300	14.9 ± 11.7	0.020
Folic (ng/ml)	300	10.16 ± 6.35	300	7.99 ± 6.17	<0.001	300	7.17 ± 4.11	<0.001
Total energy intake (kcal)	248	2581 ± 750	124	2588 ± 807	0.932	157	2548 ± 767	0.675
Protein energy %	248	11.3 ± 1.2	124	11.3 ± 1.1	0.912	157	11.4 ± 1.1	0.328
Carbohydrate energy %	248	64.1 ± 6.5	124	64.8 ± 5.5	0.284	157	64.4 ± 5.5	0.657
Fat Energy %	248	24.0 ± 4.8	124	23.5 ± 4.4	0.299	157	23.9 ± 4.5	0.879
Total Fibre (g)	248	31.9 ± 10.7	124	31.1 ± 10.8	0.510	157	32.2 ± 11.0	0.832

Physical Activity Level	228	Low (77.6%)<br>Medium (21.1%)<br>High (1.3%)	105	Low (82.9%)<br>Medium (14.3%)<br>High (2.9%)	0.230	133	Low (85.7%)<br>Medium (12.3%)<br>High (1.5%)	0.143[a]

Data shown are represented as means ± SD

P values were calculated by using the Independent t test

*P values for the differences in the means/ proportions between NGT and pre-diabetic individuals

** P values for the differences in the means/ proportions between NGT and T2D individuals

[a]P values were calculated by using the Chi Squared test

Abbreviations: CURES Chennai Urban Rural Epidemiological Study; BMI Body mass index; WC waist circumference; WHR waist to hip ratio
