# Supplementary material for: Evidence for the association between FTO gene variants and vitamin B12 concentrations in an Asian Indian population
Source: Genes Nutr. 2019 Sep 5;14:26. doi: 10.1186/s12263-019-0649-3 (PMC6728975; doi:10.1186/s12263-019-0649-3)
Supplement: Supplementary file 2 — Table S2. Interaction between the FTO-GRS and lifestyle factors on vitamin B12, folic acid, homocysteine and obesity traits. (PDF 96 kb) [file 12263_2019_649_MOESM2_ESM.pdf]

Supplementary Table 2: Interaction between the *FTO*-GRS and lifestyle factors on vitamin B12, folic acid, homocysteine and obesity traits

	BMI (kg/m^2)	WC (cm)	Vitamin B12(pg/mL)	Homocysteine (μmol/L)	Folic acid (ng/ml)
**Interaction between the GRS and carbohydrate energy (%)**	-0.08 ± 0.09	0.02 ± 0.11	1.40 ± 3.55	-0.03 ± 0.13	0.01 ± 0.10
P value	†0.387	0.882	0.694	0.83	0.952
**Interaction between the GRS and Fat energy (%)**	0.23 ± 0.12	0.18 ±0.15	0.98 ± 4.87	-0.07 ± 0.18	0.09 ± 0.14
P value	†0.052	0.225	0.841	0.709	0.539
**Interaction between the GRS and Protein energy (%)**	0.37 ± 0.50	0.77 ± 0.59	6.10 ± 19.95	0.03 ± 0.75	0.20 ± 0.58
P value	†0.451	0.196	0.76	0.968	0.728
**Interaction between the GRS and Fibre (g)**	0.08 ± 0.05	0.14 ± 1.49	1.68 ± 1.90	-0.01 ± 0.07	0.04 ± 0.05
P value	†0.081	0.925	0.376	0.898	0.503
**Interaction between the GRS and physical activity levels**	1.14 ± 1.16	-0.14 ± 1.46	23.02 ± 51.29	0.99 ± 1.93	0.46 ± 1.51
P value	†0.327	0.924	0.654	0.609	0.760

Values are beta coefficients ± standard errors.

P values were obtained by using a general linear model adjusted for age, BMI, Type 2 diabetes status, Type 2 diabetes medication and sex

† P values were obtained by using a general linear model adjusted for age, Type 2 diabetes status, Type 2 diabetes medication and sex

Abbreviations: BMI body mass index; WC waist circumference; WHR waist to hip ratio
