# Supplementary material for: Evidence for the association between FTO gene variants and vitamin B12 concentrations in an Asian Indian population
Source: Genes Nutr. 2019 Sep 5;14:26. doi: 10.1186/s12263-019-0649-3 (PMC6728975; doi:10.1186/s12263-019-0649-3)
Supplement: Supplementary file 3 — Table S3. Interaction between the FTO-GRS and dietary factors on obesity. (PDF 33 kb) [file 12263_2019_649_MOESM3_ESM.pdf]

Supplementary Table 3: Interaction between the *FTO*-GRS and lifestyle factors on obesity

	Interaction between the GRS and dietary factors on Obesity				Interaction between the GRS and physical activity levels on Obesity
	GRS * Fat energy %	GRS * carbohydrate energy %	GRS * protein energy %	GRS * fibre (g)	
Odds Ratio (95% CI)	1.096 (0.980-1.227)	0.962 (0.889-1.040)	1.113 (0.729-1.762)	1.034 (0.984-1.086)	1.026 (0.263-4.003)
P Value	0.109	0.326	0.578	0.182	0.970

Values are beta coefficients ± standard errors.

P values were obtained by using binary logistic regression adjusted for age, T2D, Type 2 diabetes medication and sex
